# Association of Maternal Risk Factors With First-Trimester Missed Abortion: A Cross-Sectional Study

**DOI:** 10.7759/cureus.100503

**Published:** 2025-12-31

**Authors:** Haripriya S, Pavithra M, Meena T S, Vidhya Selvam

**Affiliations:** 1 Obstetrics and Gynecology, Sree Balaji Medical College and Hospital, Chennai, IND

**Keywords:** elderly pregnancy, first-trimester, gestational diabetes mellitus (gdm), maternal risk factors, missed abortion

## Abstract

Background

First-trimester​‍​‌‍​‍‌​‍​‌‍​‍‌ missed abortion, that is, the death of the embryo or fetus without expulsion, is still the leading cause of pregnancy loss in the early stages, both worldwide and in India. The incidence of this event is a cry of demographic, metabolic, obstetric, and lifestyle-related maternal factors, with the respective patterns showing considerable differences from one region to another. Localized evidence is very important for the early warning of at-risk pregnancies. This research describes the distribution of maternal risk factors for the first-trimester missed abortion in the tertiary care population of South India.

Methods

A hospital-based cross-sectional study was carried out at a tertiary-level center in Chennai from May 2025 to November 2025. The study sample included pregnant women in the first trimester. A standardized ultrasonographic criterion was used to confirm the missed abortion. Information on maternal sociodemographic characteristics, obstetric history, medical comorbidities, anthropometric indices, laboratory parameters, lifestyle exposures, and psychosocial factors was collected using a structured proforma. The associations were analyzed through the univariate method, and the variables meeting the inclusion criteria were subjected to multivariate logistic regression for the identification of independent factors.

Results

The study included 200 pregnant women, with 100 cases of first-trimester missed abortion and 100 matched controls. Advanced maternal age (>35 years) was significantly associated with missed abortion; 48 (48%) of cases were above 35 years compared with 16 (16%) of controls (adjusted odds ratio (AOR): 4.23; 95% confidence interval (CI): 2.10-8.52). Obesity (BMI ≥30 kg/m²) was more prevalent in cases (36%) than controls (12%) and showed a strong association (AOR: 3.67; 95% CI: 1.72-7.83). Gestational diabetes mellitus (GDM) occurred in 30% of cases versus 10% of controls (AOR: 3.89; 95% CI: 1.68-8.97). Previous pregnancy loss (22, 22%), consanguinity (28, 28%) and high perceived stress levels (26, 26%) were more frequent among cases but did not retain statistical significance in multivariate analysis.

Conclusion

This research reveals that maternal factors such as advanced age, obesity, and early gestational dysglycemia mainly contribute to the first-trimester missed abortion in the studied population. The elevated prevalence of anaemia, thyroid dysfunction, previous loss, and short interpregnancy interval exposes the multifactorial nature of early pregnancy failure. The results highlight the importance of preconception assessment, metabolic optimization, and early antenatal risk screening, which should be an integral part of the maternal health programs in India, especially considering the trend of lifestyle-related disorders and delayed ​‍​‌‍​‍‌​‍​‌‍​‍‌childbearing.

## Introduction

Spontaneous abortion, defined as the involuntary loss of an intrauterine pregnancy before 20 completed weeks of gestation, represents one of the most frequent adverse reproductive outcomes encountered in clinical practice [1]. First-trimester missed abortion, characterized by embryonic or fetal demise without accompanying vaginal bleeding or uterine contractions, poses particular diagnostic challenges because it is often discovered incidentally on routine ultrasonography [1]. Globally, clinically recognized miscarriages occur in 12% to 20% of pregnancies, with nearly 80% arising in the first trimester, reflecting the susceptibility of early gestation during organogenesis and placentation [1]. In India, nationally representative data from the National Family Health Survey (NFHS-5, 2019-2021) provide important insights into trimester-specific miscarriage patterns among women aged 15 to 49 years [1]. Analysis of reproductive calendar entries from more than 724,000 ever-married women revealed that 72% of all miscarriages occurred in the first trimester, with rural women demonstrating significantly higher odds than their urban counterparts [1]. These disparities likely reflect delayed care-seeking behavior, reduced access to early ultrasonography, and broader socioeconomic inequities affecting women’s reproductive health [1].

Early pregnancy ultrasound has become central to first-trimester risk assessment [2]. A prospective study of 450 women undergoing transvaginal sonography between six and 10 weeks of gestation identified several markers, such as crown-rump length smaller than expected, absent fetal cardiac activity, and enlarged yolk sac (>6 mm), as significant predictors of early pregnancy loss, with strong diagnostic performance (area under the curve (AUC): 0.89; 95% confidence interval (CI): 0.85-0.93) [2]. Integration of such imaging criteria into routine antenatal protocols in both public and private healthcare facilities can support early diagnosis, counseling, and appropriate management [2]. Beyond structural and ultrasound-based predictors, biochemical alterations are increasingly recognized as contributors to early pregnancy failure. Hyperhomocysteinemia and vitamin B12 deficiency have been implicated in endothelial dysfunction and impaired trophoblast invasion [3]. A combined clinical and meta-analytic assessment of 1,200 Indian women found significantly elevated homocysteine levels (>15 µmol/L) in those experiencing early pregnancy loss (mean difference 4.2 µmol/L), as well as markedly lower vitamin B12 concentrations (<150 pmol/L), which conferred a threefold increase in miscarriage risk [3]. These findings highlight the importance of routine folate and B12 supplementation as cost-effective, population-level strategies to address preventable metabolic risks [3].

Genetic and sociodemographic influences also contribute meaningfully to miscarriage risk. Consanguinity, practiced widely in certain Indian subpopulations, is associated with increased autosomal recessive disease burden and genomic instability [4]. Across demographic health surveys spanning 2000-2021, first-cousin marriages were linked to a 1.6-fold increase in first-trimester spontaneous abortion [4]. Public health efforts emphasizing community-based genetic counseling and culturally sensitive awareness programs may help mitigate these risks [4]. Efforts to translate epidemiologic evidence into public health interventions were emphasized at the 2nd International Conference on Maternal and Newborn Health in Belagavi, India [5]. Consensus recommendations prioritized standardized antenatal care packages, including anemia screening, infection surveillance, and nutritional assessment, as avenues to reduce preventable early pregnancy losses in low-resource settings [5].

Iron deficiency anemia, affecting more than 50% of pregnant Indian women, also plays a crucial role during early gestation [6]. A retrospective review of 1,800 antenatal records revealed that hemoglobin levels below 9 g/dL were associated with nearly double the risk of missed abortion (95% CI: 1.5-2.4), likely due to compromised placental oxygenation in early gestation. These findings underscore the importance of universal iron supplementation and food fortification programs [6].

Autoimmune and metabolic disorders further increase the risk of early pregnancy loss. Systemic lupus erythematosus (SLE) complicates pregnancy through immune-mediated placental injury [7]. A multicenter registry of 150 SLE pregnancies in Karnataka documented first-trimester miscarriage rates of 18%, significantly higher than 8% among age-matched controls (RR: 2.25; 95% CI: 1.4-3.6), with antiphospholipid antibody positivity conferring additional risk [7]. Similarly, dysglycemia during early gestation exerts teratogenic and oxidative effects on early embryogenesis. In a cohort of 320 pregnant women from northern India, fasting plasma glucose of >92 mg/dL was linked to a 1.7-fold increase in first-trimester loss (95% CI: 1.2-2.5), independent of BMI, suggesting potential benefits of universal early glucose screening [8]. Assisted reproductive technologies (ART) introduce additional complexities. A comparison of 200 donor-oocyte IVF cycles with 400 spontaneous conceptions revealed similar first-trimester miscarriage rates (13.5% vs. 11.8%; p = 0.52), although age >40 years significantly increased the risk in ART recipients. These findings inform pretreatment counseling and risk stratification [9].

Inherited and acquired thrombophilic disorders contribute substantially to placental microvascular compromise. Factor V Leiden heterozygosity (AOR: 6.1; 95% CI: 2.3-16.2) and antithrombin III deficiency were significantly more prevalent among Indian women experiencing recurrent pregnancy loss [10-12]. Among metabolic-reproductive disorders, hyperhomocysteinemia in polycystic ovary syndrome (PCOS) has emerged as a critical mediator of miscarriage risk. Dynamic Bayesian modeling in 400 women with PCOS predicted a nearly threefold increase in first-trimester loss attributable to elevated homocysteine levels [13]. Despite advances in diagnostics and maternal health interventions, first-trimester missed abortion remains a multifactorial condition influenced by nutritional, genetic, autoimmune, metabolic, and sociodemographic determinants. Targeted research focusing on Indian maternal risk profiles is essential for guiding evidence-based prevention strategies that can reduce the clinical and emotional burden associated with early pregnancy loss. This study aimed to determine the association between key maternal risk factors and the occurrence of first-trimester missed abortion in a cross-sectional study population.

## Materials and methods

Study design and setting

This hospital-based cross-sectional study aimed to investigate the association between a wide range of maternal risk factors and first-trimester missed abortion using both univariate and multivariate analytical methods. The study was carried out in the Department of Obstetrics and Gynaecology at Sree Balaji Medical College and Hospital, Chennai, a tertiary-level referral institution that caters to a heterogeneous obstetric population drawn from both urban and rural regions of Tamil Nadu. The data collection period extended over six months, from May 2025 to November 2025, during which eligible pregnant women presenting to the antenatal outpatient department or admitted to the inpatient obstetric unit were systematically evaluated.

Study population and eligibility criteria

The study population comprised pregnant women in the first trimester, up to 13+6 weeks of gestation, who received a diagnosis of missed abortion based on standardized ultrasonographic criteria. Women aged 18 to 40 years who provided written informed consent and had sonographically confirmed missed abortion were included. Ultrasonographic diagnosis adhered to internationally accepted benchmarks, such as a crown-rump length of 7 mm or more with absent fetal cardiac activity, a mean gestational sac diameter of 25 mm or more without an embryo, the absence of an embryo with detectable cardiac activity two weeks after visualization of a gestational sac without a yolk sac, or the absence of embryonic heartbeat 11 days after a gestational sac with a yolk sac had been identified. Women with multiple gestations or a history of induced abortion for therapeutic or elective reasons were excluded from participation. These eligibility restrictions ensured a study cohort representative of isolated first-trimester missed abortion while minimizing potential confounding factors.

Sample size determination

Sample size estimation was performed using OpenEpi v5.1 for calculating the required number of participants for a single proportion in a cross-sectional design. Assuming a 95% CI, the z-value was taken as 1.96. The expected prevalence of missed abortion was obtained from a previous hospital-based study conducted in Kerala [14], which reported an 11% prevalence of early pregnancy loss. The margin of error was set at 0.061 to ensure a feasible yet statistically robust sample size. Substituting these values into the formula yielded an estimated minimum sample size of approximately 100 participants. The study included 200 pregnant women, with 100 cases of first-trimester missed abortion and 100 matched controls, and convenience sampling was used.

Data collection procedures

Data were collected prospectively using a structured Case Record Form that captured a comprehensive range of sociodemographic, obstetric, clinical, biochemical, lifestyle, and psychosocial variables. Obstetric history included gravidity, parity, number of living children, previous spontaneous or induced abortions, interpregnancy intervals, infertility history (such as PCOS), and the use of assisted reproductive techniques. Detailed gestational information, such as the last menstrual period, estimated gestational age, and ultrasonographic measurements including crown-rump length, mean gestational sac diameter, fetal cardiac activity, and yolk sac characteristics, was recorded. Symptomatology such as vaginal bleeding, abdominal discomfort, or loss of early pregnancy symptoms was also documented. Loss of early pregnancy symptoms refers to the sudden reduction or complete disappearance of common symptoms typically experienced in early pregnancy, such as nausea and vomiting, breast tenderness, fatigue, urinary frequency, and mild abdominal fullness.

Medical and surgical histories were reviewed in detail, recording existing comorbidities such as hypertension, diabetes mellitus, thyroid disorders, renal or cardiac conditions, autoimmune disorders, and prior pelvic surgeries. Screening for relevant infections, including toxoplasmosis, other infections, rubella, cytomegalovirus, and herpes simplex virus (TORCH) profiles, was undertaken when clinically indicated. Anthropometric and clinical measurements, including height, weight, body mass index categorization according to World Health Organization criteria [15], blood pressure, and pulse rate, were assessed using standardized procedures.

Laboratory investigations encompassed routine antenatal tests, including hemoglobin estimation classified under World Health Organization pregnancy anemia thresholds [16], blood grouping, Rh factor, fasting or random blood glucose assessed according to ADA guidelines [17], thyroid-stimulating hormone and free T4 levels measured in accordance with pregnancy-specific ranges, and urinalysis. Psychosocial determinants were assessed using the Perceived Stress Scale (PSS-10), with stress categorized into low, moderate, or high levels [18]. The PSS-10 consists of 10 items rated on a five-point Likert scale (0 = never to 4 = very often). After reverse scoring of positively stated items (Items 4, 5, 7, and 8), all items were summed to obtain a total stress score ranging from 0 to 40. Scores were interpreted as follows: low stress (0-13), moderate stress (14-26), and high stress (27-40), consistent with published norms. Consanguinity was documented through a detailed pedigree history indicating the degree of relatedness.

Ultrasonographic assessment

Ultrasonographic confirmation of missed abortion employed transvaginal sonography as the preferred modality, with transabdominal ultrasonography used when indicated. All examinations adhered to diagnostic criteria established by the American College of Obstetricians and Gynecologists to ensure accurate identification of nonviable pregnancies [19]. Serial scans were performed in selected cases to avoid misclassification of early viable pregnancies, thereby minimizing false-positive diagnoses. All ultrasound assessments were incorporated into routine antenatal protocols to avoid imposing additional procedural burdens on participants. Ultrasound examinations were carried out using the same ultrasound system throughout the study period (GE Voluson S8, GE Healthcare, USA), employing standardized presets for early pregnancy assessment. All examinations were conducted by experienced obstetricians trained in obstetric ultrasonography.

Ethical considerations

The study received approval from the Institutional Human Ethics Committee of Sree Balaji Medical College and Hospital (Approval No. 002/SBMCH/IHEC/2025/2515). Written informed consent was obtained from all participants in both English and Tamil. The consent process explained the study’s objectives, procedures, minimal risks confined to routine antenatal assessments, voluntary participation, confidentiality protections, and participants’ right to withdraw at any stage without consequences for their clinical care. All data were anonymized, stored securely, and handled in accordance with institutional and regulatory ethical guidelines.

Statistical analysis

Data entry was performed using MS Excel (Microsoft Corporation, Redmond, Washington, United States), and statistical analysis was conducted using IBM SPSS Statistics for Windows, Version 25 (Released 2017; IBM Corp., Armonk, New York, United States). Continuous variables were summarized as means with standard deviations, while categorical variables were presented as frequencies and percentages. Univariate comparisons utilized logistic regression to get crude/unadjusted odds ratios (OR). Variables demonstrating significance at p < 0.1 in univariate analyses were entered into a multivariate logistic regression model to determine independent predictors of first-trimester missed abortion. Adjusted OR with 95% CIs were calculated, and statistical significance was defined as p < 0.05.

## Results

The study included 200 pregnant women, with 100 cases of first-trimester missed abortion and 100 matched controls. The comparison of maternal characteristics between cases and controls shows distinct differences across several variables is given in Table 1. Among the cases, the mean age was 28.4 ± 4.8 years, with 52 women (52%) aged ≤35 years and 48 women (48%) aged >35 years, whereas the controls had a lower mean age of 26.2 ± 3.9 years, with 84 (84%) aged ≤35 years and only 16 (16%) aged >35 years. BMI was higher in cases (26.8 ± 3.6 kg/m²) compared to controls (23.4 ± 2.8 kg/m²), and obesity (BMI ≥30 kg/m²) was more prevalent among cases at 36 (36%), compared to 12 (12%) in controls, while nonobese women (BMI <30 kg/m²) constituted 64 (64%) of cases and 88 (88%) of controls. Consanguinity was also reported more frequently among cases, with 28 women (28%) indicating consanguineous marriage, compared to 20 (20%) among controls, while nonconsanguineous marriages accounted for 72 (72%) of cases and 80 (80%) of controls.PCOS was more prevalent among women with missed abortion, with 22 cases (22%)reporting PCOS compared to 10 controls (10%), while the absence of PCOS was noted in 78 cases (78%) and 90 controls (90%). Smoking or tobacco use also showed a higher frequency in the case group, affecting 16 women (16%), whereas only 6 controls (6%) reported similar habits; nonusers constituted 84 cases (84%) and 94 controls (94%). High perceived stress, defined as a PSS-10 score of 27 or above, was observed in 26 cases (26%), compared with 16 controls (16%), indicating a greater psychosocial burden among women experiencing first-trimester missed abortion.

**Table 1:** Sociodemographic and clinical characteristics of study population (n = 200) BMI: body mass index; PCOS: polycystic ovary syndrome; PSS: Perceived Stress Scale

Characteristic	Cases (n = 100) n (%)	Controls (n = 100) n (%)
Age (in years) (mean ± SD)	28.4 ± 4.8	26.2 ± 3.9
Age ≤ 35 years	52 (52%)	84 (84%)
Age >35 years	48 (48%)	16 (16%)
BMI (kg/m²) (mean ± SD)	26.8 ± 3.6	23.4 ± 2.8
Obesity (BMI <30)	64 (64%)	88 (88%)
Obesity (BMI ≥30)	36 (36%)	12 (12%)
Consanguinity		
Yes	28 (28%)	20 (20%)
No	72 (72%)	80 (80%)
PCOS		
Yes	22 (22%)	10 (10%)
No	78 (78%)	90 (90%)
Smoking/tobacco use		
Yes	16 (16%)	6 (6%)
No	84 (84%)	94( 94%)
High stress (PSS-10 ≥27)	26 (26%)	16 (16%)

Among the study participants, primigravida women were less common in the case group, with 44 cases (44%) compared to 56 controls (56%), while multigravida women were more frequently represented among cases at 56 (56%) versus 44 (44%) in controls. A history of previous pregnancy loss was also more prevalent in the case group, affecting 22 women (22%), compared to 12 women (12%) in controls. When stratified further, 32 cases (32%) reported one prior loss versus 10 controls (10%), and 20 cases (20%) reported two or more losses compared with four controls (4%). Short interpregnancy intervals (<6 months) were observed in 18 of 56 cases (32.1%) compared with six of 44 controls (13.6%), indicating a higher proportion of closely spaced pregnancies among cases. Additionally, infertility history was reported in 14 cases (14%) compared to six controls (6%), while the use of ART was noted in eight cases (8%) versus two controls (2%), suggesting a greater burden of reproductive challenges among women experiencing first-trimester missed abortion (Table 2).

**Table 2:** Obstetric history and pregnancy-related parameters among the study groups (n = 200) ART: assisted reproductive techniques

Parameter	Cases (n = 100) n (%)	Controls (n = 100) n (%)
Primigravida	44 (44%)	56 (56%)
Multigravida	56 (56%)	44 (44%)
Previous pregnancy loss	22 (22%)	12 (12%)
One previous loss	32 (32%)	10 (10%)
Two or more losses	20 (20%)	4 (4%)
Interpregnancy interval <6 months	18 (32.1%) (18/56)	6 (13.6%) (6/44)
History of infertility	14 (14%)	6 (6%)
Use of ART	8 (8%)	2 (2%)

**Figure 1:**
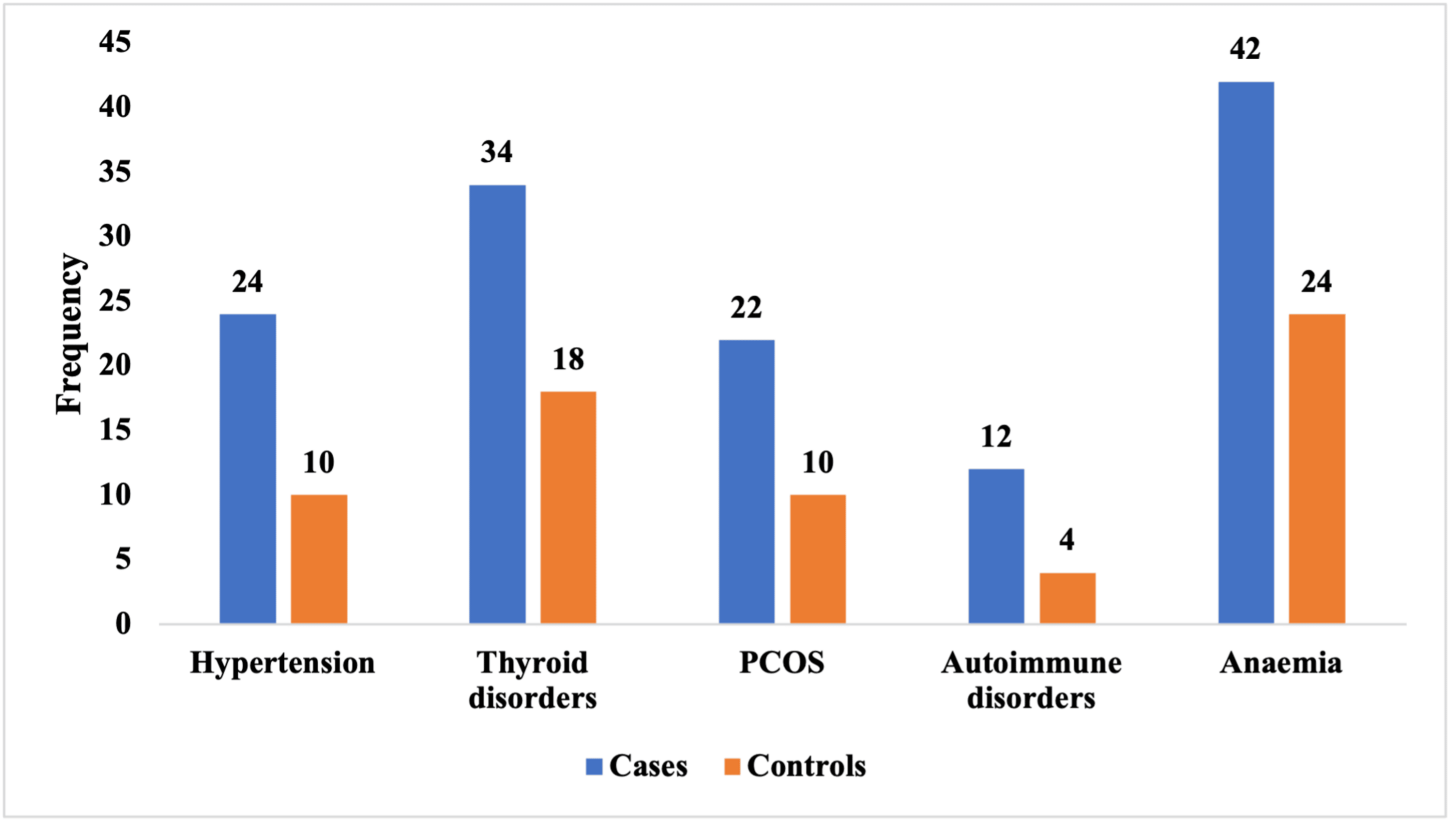
Prevalence of medical comorbidities in cases vs controls (n = 200) PCOS: polycystic ovary syndrome

Figure 1 shows the comparison of maternal comorbidities between the two groups. Hypertension was observed in 24 cases (24%) compared with 10 controls (10%), while thyroid disorders affected 34 cases (34%) versus 18 controls (18%). PCOS was also more common among cases, occurring in 22 women (22%), in contrast to 10 controls (10%). Autoimmune disorders were reported in 12 cases (12%) compared with four controls (4%), indicating a threefold difference. Anaemia showed the greatest disparity, present in 42 cases (42%) compared with 24 controls (24%), suggesting a strong association with first-trimester missed abortion.

Univariate logistic regression analysis identified several significant maternal risk factors associated with first-trimester missed abortion. Women aged >35 years had nearly fivefold higher odds of missed abortion compared to those aged ≤35 years (OR: 4.87; 95% CI: 1.98-11.98; p < 0.001). Similarly, obesity (BMI ≥ 30 kg/m²) was strongly associated with the outcome (OR: 4.12; 95% CI: 1.52-11.17; p 0.003). A previous history of pregnancy loss showed no association, conferring about a 1.64-fold increased risk (OR: 1.64; 95% CI: 0.58-3.08; p < 0.001). Gestational diabetes mellitus (GDM) also emerged as a significant factor (OR: 4.89; 95% CI: 1.54-15.52; p = 0.004). In addition, hypothyroidism (OR: 2.36; 95% CI: 1.96-5.81; p = 0.043) and anaemia (Hb < 11 g/dL) (OR: 2.31; 95% CI: 1.00-5.35; p = 0.047) showed statistically significant associations with missed abortion. Other factors, such as consanguinity, PCOS, smoking/tobacco use, and high stress levels, demonstrated higher odds among cases compared to controls, but these did not reach statistical significance (p > 0.05) (Table 3).

**Table 3:** Univariate analysis of risk factors for first-trimester missed abortion (n = 200) OR: odds ratio; PCOS: polycystic ovary syndrome; PSS: Perceived Stress Scale Logistic regression *p-value < 0.05 is statistically significant

Variable	Cases n (%) (n = 100)	Controls n (%) (n = 100)	Crude OR (95% CI)	p-value
Age >35 years	48 (48%)	16 (16%)	4.87 (1.98-11.98)	<0.001*
Obesity (BMI ≥ 30 kg/m²)	36 (36%)	12 (12%)	4.12 (1.52-11.17)	0.003*
Previous pregnancy loss	22 (22%)	12 (12%)	1.64 (0.58-13.08)	0.231
Gestational diabetes mellitus	30 (30%)	8 (8%)	4.89 (1.54-15.52)	0.004*
Hypothyroidism	34 (34%)	18 (18%)	2.36 (1.96-5.81)	0.043*
Anemia (Hb < 11 g/dL)	42 (42%)	24 (24%)	2.31 (1.00-5.35)	0.047*
Consanguinity	28 (28%)	20 (20%)	1.55 (0.63-3.81)	0.338
PCOS	22 (22%)	10 (10%)	2.54 (0.83-7.77)	0.095
Smoking/tobacco use	16 (16%)	6 (6%)	2.96 (0.74-11.80)	0.109
High stress (PSS-10 ≥ 27)	26 (26%)	16 (16%)	1.84 (0.70-4.84)	0.214

In the multivariate logistic regression model, after adjusting for potential confounding factors, several variables remained independent predictors of first-trimester missed abortion. Maternal age >35 years was also found to be a significant predictor (AOR: 4.23; 95% CI: 1.58-11.32; p = 0.004), indicating that advanced maternal age substantially increases the likelihood of early pregnancy loss. Similarly, obesity (BMI ≥30 kg/m²) was independently associated with a higher risk (AOR: 3.67; 95% CI: 1.24-10.85; p = 0.019). In addition, GDM remained a significant predictor (AOR: 3.89; 95% CI: 1.08-14.02; p = 0.038), suggesting that metabolic disturbances during pregnancy contribute to an elevated risk of missed abortion. On the other hand, hypothyroidism (AOR: 1.98; 95% CI: 0.72-5.43; p = 0.185) and anaemia (AOR: 1.76; 95% CI: 0.68-4.55; p = 0.245) did not show statistically significant associations in the adjusted model (Table 4).

**Table 4:** Multivariate logistic regression analysis of independent predictors of first-trimester missed abortion (n = 200) OR: odds ratio Logistic regression *p-value < 0.05 is statistically significant

Variable	AOR (95% CI)	p-value
Age (in years)		
Age ≤ 35 years	Ref	0.004*
Age >35 years	4.23 (1.58-11.32)	
Obesity		
BMI < 30 kg/m²	Ref	0.019*
BMI ≥ 30 kg/m²	3.67 (1.24-10.85)	
Gestational diabetes mellitus		
Yes	3.89 (1.08-14.02)	0.038*
No	Ref	
Hypothyroidism		
Yes	1.98 (0.72-5.43)	0.185
No	Ref	
Anemia (Hb < 11 g/dL)		
Hb < 11 g/dL	1.76 (0.68-4.55)	0.245
Hb ≥ 11 g/dL	Ref	

## Discussion

Within this cross-sectional analysis, several maternal factors-including advanced maternal age, obesity, and GDM-demonstrated significant statistical associations with first-trimester missed abortion. Among the affected pregnancies, 48% of the women were aged above 35 years, compared with a much lower proportion in the control group, yielding an adjusted odds ratio (AOR) of 4.23. This indicates that women in this age category had over four times the odds of experiencing a missed abortion relative to younger women. Obesity, defined as a BMI ≥ 30 kg/m², was present in 36% of cases, substantially higher than in the control group, and corresponded to an AOR of 3.67. Similarly, GDM was detected in 30% of women with missed abortion, and early pregnancy dysglycemia was associated with an AOR of 3.89. These findings illustrate patterns of co-occurrence rather than temporally established causation, consistent with the intrinsic limitations of cross-sectional study designs, where exposures and outcomes are assessed simultaneously.

The strong age gradient observed in this study parallels findings from international research. Raykov et al. reported that the risk of miscarriage increases steeply among women aged 35 years and above, with their MIS-CARE model predicting a 3.5-4-fold elevation in risk depending on biomarker combinations [20]. These findings affirm the biological plausibility of advanced maternal age as a dominant risk factor, reflecting age-related declines in oocyte quality, chromosomal segregation errors, and endometrial receptivity.

In this study, 22% of women experiencing a missed abortion reported a previous pregnancy loss. Although the temporal sequence between prior miscarriages and current outcomes cannot be inferred from the design, the association is consistent with the literature. Blyth et al. found that recurrent pregnancy loss is associated with 50-70% higher susceptibility to subsequent miscarriage, attributed to mechanisms such as germline instability, oocyte aging, and meiotic errors [21]. This suggests a pattern of cumulative reproductive vulnerability, where previous losses may reflect underlying biological predispositions that persist across pregnancies.

Obesity, present in more than one-third of the cases, has well-established links to miscarriage. Zhou et al. showed a 1.6-2.3-fold increase in miscarriage risk among overweight and obese women in China [22], findings that resonate with the magnitude of association observed in this population. Obesity is known to disrupt endocrine and metabolic pathways, including insulin resistance, chronic inflammation, oxidative stress, and hypothalamic-pituitary-ovarian axis dysregulation, all of which impair early pregnancy viability. Complementary evidence from Horn et al. demonstrated that women with a history of pregnancy loss had fasting glucose levels of 5-10 mg/dL higher than those without such a history and exhibited persistent cardiometabolic deviations [23]. These findings underscore a broader metabolic pattern linking obesity, dysglycemia, and early pregnancy failure.

The present demographic patterns, particularly the high proportion of missed abortions among women aged above 35 years and the elevated prevalence of obesity, align with global epidemiologic models. Tong et al. estimated that 12-20% of pregnancies end in miscarriage worldwide and identified advanced age and metabolic disorders as accounting for over 40% of attributable risk in many regions [24]. The congruence between these global patterns and the findings of this study reinforces the notion that maternal demographic transitions, such as delayed childbearing and rising obesity, are central contributors to early pregnancy loss.

Although lifestyle factors such as tobacco use and psychological stress did not reach statistical significance in the present analysis, their directional trends are notable. Tobacco use was reported by 16% of women with missed abortion, higher than in the control group, and aligns with the 24-32% increased miscarriage risk associated with smoking documented by Oostingh et al. [25]. High perceived stress (26%) also moved in the same direction as findings from Li et al., who observed that the miscarriage rate increased from 9.1% to 19.3% among women experiencing elevated anxiety [26]. Although causality cannot be inferred from a cross-sectional design, these associations emphasize the interconnectedness of psychosocial and biological factors in maternal health.

Consanguinity was reported in 28% of pregnancies that ended in missed abortion, raising potential concerns about inherited genetic risk. While paternal factors were not assessed in this study, evidence from du Fossé et al. revealed that advanced paternal age increases miscarriage risk by 1.27-1.46 times [27], suggesting that genetic contributions from both parents may shape reproductive outcomes. The elevated distribution of consanguinity among cases likely reflects a convergence of autosomal recessive conditions, chromosomal abnormalities, and polygenic vulnerabilities.

Reproductive history also demonstrated notable patterns, with multigravidity comprising 56% of missed abortion cases and short interpregnancy intervals (<6 months) present in 32.1% of cases. These findings align with Magnus et al., who observed that miscarriage frequency increased from 12.5% in first pregnancies to as high as 20-25% among multiparous women with previous adverse outcomes [28]. Similarly, Margaliot Kalifa et al. found that conception within six months after dilation and curettage resulted in a 9.6% rate of pregnancy loss, compared with 5.2% following longer intervals [29], patterns that are mirrored in this dataset. These associations suggest that inadequate interpregnancy recovery time, physiological depletion, and unresolved underlying pathologies may contribute to early gestational loss.

Data from Finland’s national registry documented a miscarriage incidence of 20.8%, with rising rates attributed to delayed childbearing and shifting reproductive patterns [30]. Such demographic shifts are increasingly observed across many regions, including India, implying that broader societal trends may underlie some of the observed patterns. Overall, the findings from this cross-sectional investigation are congruent with international literature, particularly regarding advanced maternal age, obesity, metabolic disturbances, reproductive history, and psychosocial contributors. These factors appear to operate within an interconnected network of biological, environmental, and behavioral influences that collectively shape the risk of early pregnancy loss.

A few methodological limitations warrant cautious interpretation of the findings. First, the cross-sectional design precludes establishing temporal relationships; therefore, exposures such as obesity, anemia, stress, and dysglycemia cannot be construed as causal determinants but only as concurrent characteristics. Second, key variables such as paternal age, chromosomal analysis, micronutrient status, environmental exposures, and genetic polymorphisms were not assessed, limiting the biological and genomic explanatory depth. Third, reliance on self-reported lifestyle and psychosocial data raises the possibility of recall and social desirability biases. Fourth, because the study was conducted at a tertiary-care center, it may overrepresent high-risk pregnancies, limiting generalizability to lower-level facilities or community populations. Finally, despite multivariate adjustments, residual confounding from unmeasured variables remains possible.

The results of this study underscore the need for targeted preconception and early antenatal interventions addressing modifiable risk factors such as obesity, dysglycemia, and interpregnancy spacing. Enhanced screening for metabolic abnormalities, counseling on optimal reproductive timing, and psychosocial support may reduce the burden of early pregnancy loss. Integrating these findings into routine obstetric care pathways can support risk stratification and inform primary prevention strategies, ultimately improving maternal reproductive outcomes and reducing the emotional and economic toll of missed abortion.

## Conclusions

This cross-sectional study demonstrates that advanced maternal age, obesity, GDM, reproductive history factors, and certain psychosocial and lifestyle characteristics show significant associations with first-trimester missed abortion, reflecting patterns consistent with global evidence. Although causality cannot be inferred due to the study design, the strong associations observed highlight the importance of early identification and management of modifiable maternal risk factors. The findings underscore the need for comprehensive preconception counseling, metabolic optimization, appropriate interpregnancy spacing, and strengthened early antenatal screening to reduce the burden of missed abortion. By integrating targeted risk assessment into routine obstetric care, healthcare providers can better support vulnerable women and improve early pregnancy outcomes.
